# Characterization of *Microcystis* (Cyanobacteria) Genotypes Based on the Internal Transcribed Spacer Region of rRNA by Next-Generation Sequencing

**DOI:** 10.3389/fmicb.2018.00971

**Published:** 2018-05-15

**Authors:** Da Huo, Youxin Chen, Tao Zheng, Xiang Liu, Xinyue Zhang, Gongliang Yu, Zhiyi Qiao, Renhui Li

**Affiliations:** ^1^National Demonstration Center for Experimental Aqua-ecology and Aquaculture Education, Department of Fisheries Sciences, Tianjin Agricultural University, Tianjin, China; ^2^Key Laboratory of Algal Biology, Institute of Hydrobiology, Chinese Academy of Sciences, Wuhan, China

**Keywords:** cyanobacteria, Yuqiao reservoir, Haihe River, high throughput sequencing, ITS region

## Abstract

*Microcystis* is one of the most common and dominant bloom-forming cyanobacteria in freshwater worldwide. The method for genotype detection based on traditional molecular cloning is expensive and time consuming and generates a limited number of sequences. In this study, a high-throughput sequencing (HTS) method was developed to detect the internal transcribed spacer (ITS) regions between 16S and 23S rRNA region of *Microcystis* populations along a typical water system in Yuqiao Reservoir–Haihe River in Tianjin, northern China. A total of 629,341 reads were obtained and clustered into 2005 operational taxonomic units (OTUs). Analysis of alpha diversity indices showed that the Haihe River is more diverse than Yuqiao Reservoir. In general, the two water areas exhibit a clear differentiation pattern in OTU abundance, sharing genotypes from a small part of Yuqiao Reservoir with those in the Haihe River. Phylogenetic analysis further indicated the possible flexible evolution of *Microcystis* genotypes occurring in the research areas. This study provides the first exhaustive description of HTS method for detection of ITS region to evaluate *Microcystis* intra-species diversity and relationship.

## Introduction

Cyanobacteria are prokaryotes regarded as the oldest oxygenic photosynthetic organisms on earth (Hess, 2011; Wittmann and Liao, 2016). With a broad adaption in most living habitats, especially, prominent standing capacity for extreme environment (Cristiana et al., 2013; Klanchui et al., 2017), cyanobacteria are widely distributed worldwide (Whitton, 2012; Visser et al., 2016). They are abundant components of aquatic ecosystems, particularly in eutrophicated waters, and may form blooms in water bodies (Chislock et al., 2013). Cyanobacterial blooms have become a serious global issue in fresh water lakes, rivers, and reservoirs. Bloom-forming cyanobacteria can form scums and odorous compounds and produce toxic metabolites, such as microcystins (MCs) (Codd et al., 2005; Paerl et al., 2015). A combination of multiple biological, physicochemical, and climatic factors causes the development of harmful cyanobacterial blooms. Eutrophication of water bodies is the main factor promoting development of cyanobacterial bloom in the past decades (Bonilla et al., 2012; O’neil et al., 2012). However, global climate change and the rise of carbon dioxide concentration have been regarded as important factors that trigger the extensive development of cyanobacterial blooms as well (Paerl and Huisman, 2008; Verspagen et al., 2014). MCs are hepatotoxins and tumor promoters that are mainly produced by *Microcystis*, a unicellular colony-forming cyanobacterial group. *Microcystis*-dominated blooms have largely occurred worldwide, Therefore, numerous studies have been focused on *Microcystis*-dominated blooms and their harmful effects (Li et al., 2012; Srivastava et al., 2012).

The genetic diversity, composition, and spatiotemporal dynamics of cyanobacteria populations have been widely studied to further understand their proliferation. Sequencing of a specific gene marker provides a way to track the dynamic variation in cyanobacterial communities (Stucken et al., 2009). Because of its wide distribution and harmful influence, the genetic characteristics of *Microcystis* have been studied extensively. (Nübel et al., 1997; Zhang et al., 2009; Tan et al., 2010; Xu et al., 2010). *Microcystis* was shown to be highly diverse at the intra-species level not only among geographically distant ecosystems but also during bloom proliferation in localized freshwater environments (Kataoka et al., 2013). The internal transcribed spacer (ITS) region of 16S-23S rRNA is an effective molecular marker for characterization of *Microcystis* intra-species diversity (Yoshida et al., 2008; Moreira et al., 2016), and several studies used ITS region as gene marker to examine the cyanobacterial community structures (Otten et al., 2015). Compared with the Oligotypes of 16S rRNA gene used in Lake Erie (Berry et al., 2017), ITS provide a higher genetic resolution of *Microcystis* strains (Briand et al., 2009; Xu et al., 2010; Lemaire et al., 2012; Liu et al., 2016). However, Zhu et al. (2012) indicated that no significantly dominant genotypes existed in Xinghu Pond, a small eutrophic pond in central China, partially consistent with the finding by Pobel et al. (2012) who found no spatial ITS heterogeneity of each sampling date in a french shallow lake. These conflicting results implied that complex circumstances and the multiple factors may drive the genetic composition of *Microcystis* populations in water system. Recently, researchers sequenced cyanobacterial ITS region by 454 pyrosequencing, and found eight *Microcystis* genotypes in these sequences. Even using pyrosequencing method, the sequencing depth is still not enough to uncover the high diversity of *Microcystis*, so that several PCR sequences could not be found in these eight OTUs (Otten et al., 2017). The primers specific for *Microcystis* is needed in these ecological studies.

The development of whole genome based taxonomic system should be the ideal goal for studying cyanobacterial systematics and diversity (Thompson et al., 2013; Soo et al., 2014; Walter et al., 2017). For elucidating the diversity in a specific cyanobacterial group such as *Microcystis*, analyzing a single gene is still the most convenient way. By using molecular cloning method, Sabart et al. (2009) found that most *Microcystis* genotypes seem to be ubiquitous. Nevertheless, low efficiency of this traditional cloning method causes difficulty in identifying the comprehensive diversity of *Microcystis*. The limited numbers of clones may lead to an underestimated or biased evaluation of *Microcystis* genotypes. With the development of high-throughput sequencing (HTS) techniques, current methods enable researchers to achieve faster and less costly genomic characterizations of cyanobacterial strains and raise additional information about their associated microorganisms (Danillo et al., 2017). Several studies used deep targeted sequencing as a powerful tool to detect the microbial community structure and cyanobacterial composition during cyanobacterial bloom period (Chen et al., 2011; Bertos-Fortis et al., 2016; Parulekar et al., 2017), And by improving the resolution of gene marker sequencing (Ruegger et al., 2014), Next-generation sequencing targeting the 16S rRNA gene now allows the comprehensive investigation of bacterial community composition with coverage far beyond that of previous clone libraries (Mackey et al., 2017). The effectiveness or advantage of ITS region-targeted HTS in detecting of *Microcystis* intra-species diversity remains not clear.

Yuqiao Reservoir, the drinking water sources of Tianjin City, China, supports more than one million people’s water usage. Yuqiao Reservoir is at the upper part of Haihe River flowing through the Tianjin city. The proliferation of *Microcystis* threats both Yuqiao Reservoir and downstream Haihe River (Huo et al., 2018). However, the evaluation and high-frequency monitoring of cyanobacterial blooms in Yuqiao Reservoir-Haihe River system remain uncharted. Even two water areas are connecting each other, they are shown to have different levels of eutrophication. The investigation on the diversity of *Microcystis* between these two waters will help to understand the selective mechanism on *Microcystis* under different eutrophication levels. This study aims to characterize *Microcystis* genotypes based on the ITS region of rRNA through next-generation sequencing. To comprehensively discover the potential diversity of *Microcystis* ITS region, we designed new primers based on reported sequences that are currently available in public databases. We collected water samples from cyanobacterial blooms along the Yuqiao–Haihe water system in Tianjin, northern China. Deep targeted sequencing was performed on these *Microcystis*-based blooms. The study was expected to provide information on discussing the feasibility of HTS in studying *Microcystis* ITS region diversity.

## Materials and Methods

### Sampling Strategy

**FIGURE 1:**
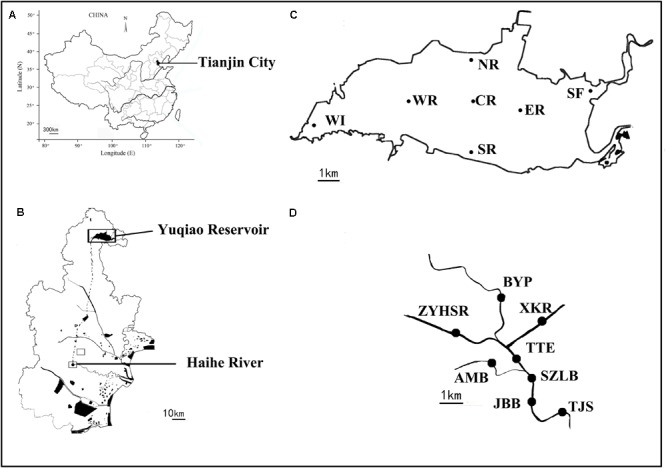
Distribution of sampling sites between two water areas. Dotted line shows the underground connecting channel between two water areas. Maps of **(A)** China, **(B)** Tianjin city, **(C)** Yuqiao reservoir, and **(D)** Haihe River. Adapted from Huo et al. (2018).

Surface water samples from 15 sites in Yuqiao Reservoir and Haihe River were collected in September 2016. The locations of the sampling sites are shown in **Figure 1**. Dissolved oxygen, water temperatures (WT), salinity (SAL), and pH were measured using YSI multi-parameter detector (YSI, United States). Chlorophyll a, total nitrogen (TN), total phosphorus (TP), chemical oxygen demand, ${\mathrm{NH}}_{4}^{+}$, and ${\mathrm{NO}}_{2}^{–}$ were analyzed according to standard methods (Jin and Tu, 1990). Water (500 ml) was filtered through a 0.22 μm filter (Millipore, United States) for HTS at each sampling sites. The filtrate was stored at -80°C until further analysis.

### DNA Extraction and Library Construction

DNA was extracted from each filtered membrane using the PowerWater DNA isolation kit following the manufacturer’s manual (Mo Bio Laboratories, Inc., Carlsbad, CA, United States). DNA concentration was evaluated using Qubit 2.0 fluorometer (Life Technologies Japan Ltd., Tokyo, Japan). We designed a specific primer with a suitable length for amplification due to limited read length on Illumina Miseq^TM^ platform. We designed the forward primer HTSITS_F as 5′ -TACACG- ACGCTCTTCCGATCTCTAG(barcode)AAGGGAGACCTAATTCRGGTA-3′, and used the reverse primer ITS_R as 5′-TGGAGTTCCTTGGCACCCGAGAA- TTCCATAGCCTCTGTGTGCCTAGGTATCC-3′ (Iteman et al., 2000). Both primers contain an Illumina adapter region for sequencing on the Illumina Miseq^TM^ platform. A 6 bp barcode in the forward primer was used to distinguish each sample. And the length of PCR products was about 550 bp. We checked the sensitivity of primers by using different cyanobacterial species as template for amplification. The result of primer specificity test is shown in **Supplementary Table S1**.

### Processing of OTUs

The quality filtering and processing of raw data were performed with cut adapt (Martin, 2011), PEAR (Zhang et al., 2014), and Prinseq (Schmieder and Edwards, 2011). We used Uchime to check and remove chimeras (Edgar et al., 2011). All samples were assigned using UPARSE method at 97% cut-off (Edgar, 2013). A Venn diagram was constructed to reflect the common OTU and unique OTU numbers in all samples using R package Venn Diagram. To further examine the specificity of primers that we designed, we used Online BLAST (Lobo, 2012) search tool to assign the taxonomy of all OTUs with the NT database of NCBI. Sequences with a similarity of >90% and coverage of >90% were considered as identified or interpreted as unclassified if otherwise. A summary of taxonomic result is shown in **Supplementary Table S2**.

### Phylogenetic Analyses

A phylogenetic tree was constructed using the top 20 abundantly represented sequences of OTUs in this study and 100 other reported representative sequences. Sequences were summarized by Song et al. (2015). All sequences were aligned with Bioedit (version 7.2.6.1) (Hall, 1999), and a neighbor-joining tree (Saitou and Nei, 1987) was constructed using MEGA (version 7.0) (Kumar et al., 2008). Evolutionary distances were computed using the Kimura 2-parameter method. Bootstrap trial was set to 1000. The final tree figure was annotated using R package ggtree (Yu et al., 2017).

### Multidimensional Analyses and Statistics

All statistical analyses were performed in the R environment using VEGAN (Dixon, 2017) and grid (Wegener et al., 2009) packages. Results were presented as mean values ± standard deviation. Alpha diversity was measured by (Quantitative Insights Into Microbial Ecology) QIIME procedure (Caporaso et al., 2010). OTU network was analyzed with QIIME and plotted using the R package Igraph (Csardi and Nepusz, 2006). We selected the OTUs with >1% abundance for the network analysis. Weighted Unifrac and Bray–Curtis Matrix were calculated with vegan package and plotted with pheatmap package. The correlation between environmental factors with OTUs was measured with Pearson’s relative indices (data shown in **Supplementary Table S3**). Detrended correspondence analysis (DCA) was performed to determine the appropriate type of model for direct gradient analysis (Jongman et al., 1995). The environmental factors were transformed with log10 (OTU#+1), and OTU abundances were transformed with Hellinger transformation (Legendre and Gallagher, 2001). DCA results show that a Unimodal model would be suitable for further analyses. Finally, canonical correlation analysis (CCA) was performed with Vegan package.

### Data Available

The ITS amplicon sequences have been deposited as dataset SRP127241 in the sequence read archive (SRA) of the National Center for Biotechnology Information (NCBI).

## Results

### Sequencing Results and Alpha Diversity Analysis

**FIGURE 2:**
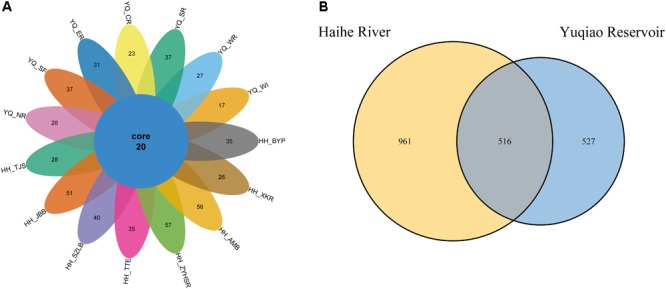
Venn diagram of OTU numbers in 15 samples. Cluster at 97% cut-off. **(A)** Individual samples. **(B)** Samples merged into groups.

**FIGURE 3:**
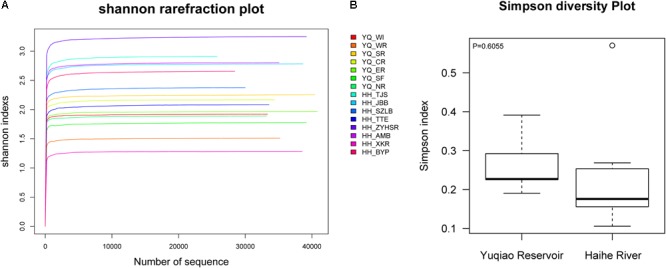
Plots of alpha diversity analyses of sequences. **(A)** Shannon rare fraction curves. **(B)** Box plot of Simpson diversity.

After quality control, we evaluated the distribution of all sequences, as shown in **Supplementary Figure S1**. All of the sequences obtained were ∼360 bp-long. The singletons were removed for further analyses. A total of 629,341 reads were obtained. OTU numbers for each sample are listed in **Table 1**. HH_ZYHSR exhibited the highest OTU number of 664, whereas YQ_WI possessed the lowest OTU number of 245. The average OTU number in Haihe River was 497.6 ± 123.3, which was significantly higher than that in Yuqiao Reservoir (325.9 ± 59.2). The Venn diagram in **Figure 2A** illustrated the common and unique OTUs in each sample. Among all samples, 20 OTUs commonly existed. HH_ZYHSR and HH_WI demonstrated the highest and lowest number of unique OTUs of 57 and 17, respectively. In two water areas, 516 common OTUs existed, and Haihe River contained double the number of unique OTUs as that of Yuqiao Reservoir (**Figure 2B**). From both views of sequencing depth and genotype numbers, HTS-based detection displayed an evidently higher efficiency than traditional library cloning method (**Table 2**). Shannon rarefaction plots indicated that most samples from the Haihe River showed higher genetic diversity than those from the Yuqiao Reservoir (**Figure 3A**). Simpson diversity plot also showed higher diversity in the Haihe River than that in the Yuqiao Reservoir (**Figure 3B**).

**Table 1:** Detailed information of sequencing result and diversity indices.

Sample	Seq num	OTU	Shannon	ACE	Chao1	Coverage	Simpson
HH_AMB	35082	508	2.801632	793.818389	771.129412	0.993957	0.155512
HH_BYP	28440	540	2.655934	1105.595904	849.141304	0.991596	0.182228
HH_JBB	38661	533	2.78349	1073.375118	816.453608	0.993922	0.156036
HH_SZLB	30003	506	2.376125	803.330239	709.584071	0.992834	0.238113
HH_TJS	25791	588	2.909642	891.559234	854.342857	0.990811	0.169032
HH_TTE	33573	371	2.082338	664.749925	520.013699	0.995592	0.268606
HH_XKR	38539	271	1.284971	552.733938	398.258621	0.996834	0.570362
HH_ZYHSR	39169	664	3.249824	1198.519414	983.918919	0.993183	0.105657
YQ_CR	34386	328	2.169507	824.480111	562.491228	0.995231	0.190352
YQ_ER	40823	309	1.968443	686.566003	488.40678	0.996424	0.225406
YQ_NR	33220	288	1.889695	809.701961	484.981132	0.995635	0.229471
YQ_SF	39158	388	1.774099	881.712147	589.658228	0.995429	0.35491
YQ_SR	40429	418	2.253835	833.221758	641.441558	0.995399	0.22677
YQ_WI	33344	245	1.920372	567.094033	406.333333	0.996371	0.225656
YQ_WR	35207	305	1.510009	670.809446	456.681818	0.995967	0.391206

**Table 2:** Summary of papers related to *Microcystis* ITS genotypes detecting methods.

Water body	Country	Method	Sequences	Dissimilarity	Genotypes	Reference
Grangent Reservoir	France	Library cloning	784	One mutation	306	Briand et al., 2009
Loire River	France	Library cloning	950	One mutation	375	Sabart et al., 2009
Qinhuai River	China	Library cloning	546	One mutation	230	Xu et al., 2011
Xing Pond	China	Library cloning	563	One mutation	320	Zhu et al., 2012
Taihu-Chaohu	China	Library cloning	84	One mutation	15	Cai et al., 2012
Erhai Lake	China	Library cloning	800	One mutation	473	Song et al., 2015
**Haihe-Yuqiao**	**China**	**High-throughput sequencing**	**629341**	**3% of sequences**	**2005**	**This study**

### Beta Diversity Analysis

**FIGURE 4:**
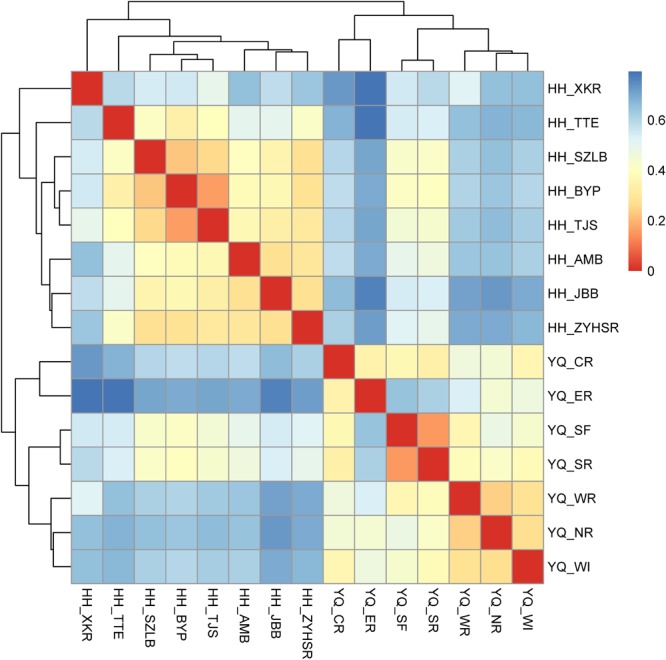
Heatmap of weighted Unifrac distance. The color of square shows the distance between each two samples. The range of blue to red corresponds to far to close distance.

**FIGURE 5:**
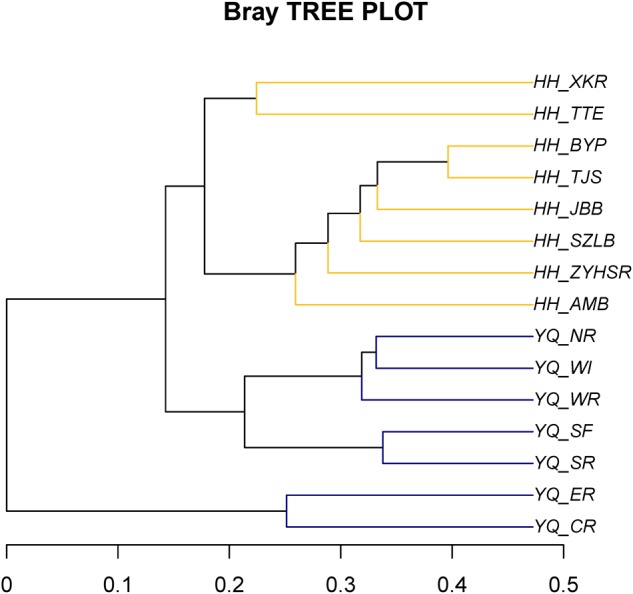
Bray–Curtis distance tree. The matrix was measured using Bray–Cutis algorithm. High similarity samples were gathered into the same branch.

**FIGURE 6:**
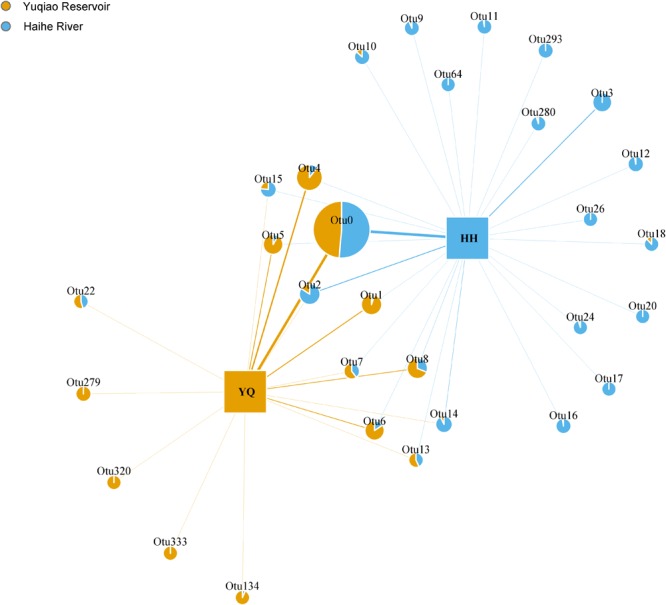
OTU network plots. Different colors show the proportion of abundance in different groups. The area of each edge shows different abundance of each OTU, and those with an abundance of >1% were included in the plot.

Weighted Unifrac distance heatmap showed that samples from the two water areas could be easily separated into two groups (**Figure 4**). Internal distance was close between Haihe River and Yuqiao Reservoir, with the two samples YQ_SF and YQ_SR as more closely related to the samples in Haihe River. The closest crossed group distance was found between YQ_SR and HH_BYP. Bray–Curtis-based distance tree also demonstrated clearly separated matrices between two water areas (**Figure 5**). HH_XKR and HH_TTE gathered into one group, whereas HH_BYP, HH_TJS, HH_JBB, HH_SZLB, HH_ZYHSR, and HH_AMB gathered into another. For the samples in Yuqiao reservoir, YQ_NR, YQ_WI, and YQ_WR were in one group, and YQ_SF and YQ_SR clustered together. YQ_ER and YQ_CR were distant from other samples. Clustered OTUs were measured by network analysis. **Figure 6** illustrates that Otu0 was the most abundant in two water areas. This OTU network displayed a clear separation between Yuqiao Reservoir and Haihe River.

### Phylogenetic Analysis

To further understand the genetic characters of ITS region, we compared the genotypes obtained in this study with those in previous studies. We constructed a neighbor-joining tree using representative sequences from top 20 abundant OTUs and the 100 previous sequences downloaded from NCBI.

**FIGURE 7:**
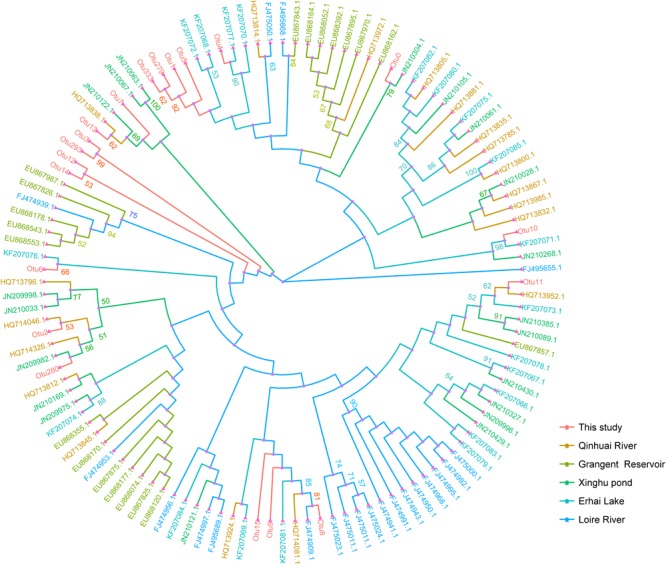
Neighbor-joining tree constructed with *Microcystis* ITS genotypes in this study and other available data. Top 20 abundantly represented sequences of OTUs in this study and 100 other publicly available data. The evolutionary distances were computed using the Kimura two-parameter method. Different color of branches shows different study areas. Bootstrap values greater than 50% are indicated on the tree.

The tree displayed no significant geographic distribution pattern (**Figure 7**). The dominant genotype Otu0 closely clustered with JN210304.1, which was detected in Xinghu pond, Wuhan, China (Zhu et al., 2012). Otu279, Otu33, Otu1, and Otu5 in this study were clustered together in a group, whereas Otu3, Otu293, Otu12, and OTu14 were clustered in another, indicating that these genotypes may possess regional characters that are unique from those obtained in previous studies. Interestingly, the results from phylogenetic and network analyses showed that most OTUs from Group 1 (Otu279, Otu1, and Otu5) dominated in Yuqiao reservoir but were rare in Haihe River. Conversely, Group 2 OTUs (Otu3, Otu293, Otu12, and Otu14) were mostly found in Haihe River but were rare in Yuqiao reservoir.

### Relationship Between OTU Abundance and Environmental Factors

**FIGURE 8:**
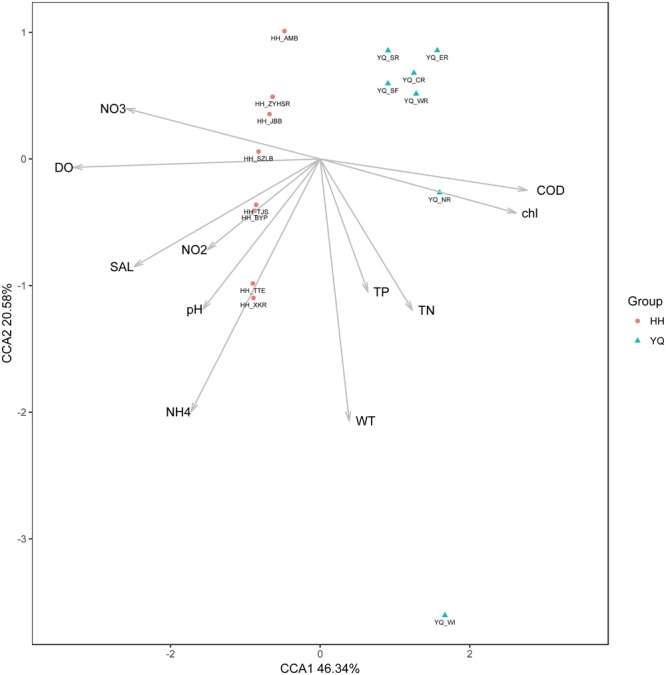
Canonical correspondence analysis showing the relationships between environmental factors and the relative abundance of OTUs.

Environmental parameters revealed that Yuqiao Reservoir had a significantly higher eutrophication level than Haihe River, as shown in the result of Huo et al. (2018). (TSI Value in Yuqiao Reservoir: 64.94 ± 2.46 and in Haihe River: 50.02 ± 10.78) Pearson correlation coefficient was calculated to evaluate the relationship between OTU abundance and environmental factors (Results in **Supplementary Table S3**), and CCA was subsequently conducted to show all their correlations. The CCA ordination showed that OTU abundance was strongly affected by environmental factors (**Figure 8**). In general, samples from Haihe River clustered together on CCA plot and could be differentiated from samples in Yuqiao Reservoir. They were positively related to DO, SAL, and different forms of dissolved nitrogen. The samples in Yuqiao Reservoir were more discrete and most samples were positively related to TN, TP, and WT. The specific OTU genotypes preferred various environmental conditions. The dominant OTU genotype Otu0 was positively and significantly affected by TN and TP (*P* < 0.05). WT, pH, and ${\mathrm{NO}}_{2}^{–}$ also contributed to the distribution of Otu0, though not significantly (*P* > 0.05).

## Discussion

Using molecular biological approach to investigate the spatiotemporal dynamics of *Microcystis* populations has become an important tool for understanding *Microcystis* population genetics and the spatial development of a bloom in a water system (Kardinaal et al., 2007; Briand et al., 2009; Bozarth et al., 2010). Such molecular approaches have largely advanced from denaturing gradient gel electrophoresis and clone sequencing to HTS by next-generation sequencing. The present study is, to the best of our knowledge, the first application of HTS in examining *Microcystis* intra-species diversity on the ITS region. Previous works in detecting *Microcystis* genotype with library cloning could only provide dozens to thousands of sequences (**Table 2**). By defining one base pair divergence as a new genotype, these previous studies using the cloning-based method could identify up to hundreds of genotypes in a water body, such as 618 genotypes from 2337 ITS sequences in Lake Taihu—the maximum number of ITS genotypes so far (Liu et al., 2016). The HTS result of the present study obtained 629,341 sequences in Yuqiao-Haihe water system. Although 97% cut-off was set as the OTU threshold, the thousands of OTUs identified in this study after removing singletons indicated that the *Microcystis* populations were extremely diverse at the ITS region. Therefore, assigning 97% cut-off in ITS similarity as an OTU provides a highly reasonable definition to reveal divergence among each OTU and sample site. We verified that HTS brings much bigger sampling size than traditional library cloning method.

The comparison in the ITS genetic diversity of *Microcystis* blooms between the two water areas showed that Haihe River is more diverse than Yuqiao Reservoir. The environmental parameters detected already showed that Yuqiao was more eutrophicated than Haihe River. Thus, the genetic diversity of the ITS genotypes was negatively correlated with eutrophication level, which was very similar to the result obtained in Erhai Lake by Song et al. (2015). Yuqiao Reservoir is a semi-closed reservoir, and water from Yuqiao Reservoir flows into Haihe River as partial water resource. The different water resource flow-in may lead to an increased *Microcystis* intra-species diversity. Briand et al. (2009) reported that the variation in the dominant genotypes was transformational during a bloom and tracing the dominant species could also provide a way to find the seed source triggering the blooms (Xu et al., 2011). Through beta diversity analyses, a clearly separated relationship between two water areas was displayed. Most sites demonstrated different abundant characters between two water areas, but the samples from two sites in Yuqiao (YQ_SF and YQ_SR) were revealed to be closely related to those in Haihe River, indicating that *Microcystis* populations in these two sampling sites may contribute and adapt to the environmental conditions in Haihe River. Thus, these genotypes were maintained during water exchange in the two water areas. To better understand the factors driving the differentiation of genotypes, we investigated the relationship between environmental factors with different samples and genotypes. As shown in the results, the only dominant genotype Otu0 was affected by TN and TP (**Figure 8**), implying that TN and TP play an important role in the formation of dominant genotypes. The proportion of Otu0 abundance was almost 1:1 between two water areas, and the Otu0 point on CCA plot was almost located at the perpendicular bisector of the samples from the two water areas, implying that the dominance of Otu0 was attributed to its great adaptive capacity in both waters.

Phylogenetic analyses indicated that no significant spatial distribution was found in the pattern of dominant genotypes, which was similar to the result in Erhai Lake, Yunnan, China (Song et al., 2015). Interestingly, several dominant OTUs were clustered together in the phylogenetic tree (Group I and Group II), and these OTUs were mostly found in Yuqiao Reservoir but rare in Haihe River. Such clustering pattern indicated that these *Microcystis* genotypes exhibit their own evolutionary adaption by developing a group of mutants to satisfy the variation of habitats. But this kind of adaption could be effective only in a small region. In this study, these genotypes could not be adapted to environment in Haihe River. Thus, it was difficult to develop the Microcystis genotypes in Yuqiao Reservoir into Haihe River along the water flow. High genomic plasticity was reported for *Microcystis* (Kaneko et al., 2007; Frangeul and Al, 2008), and *Microcystis* species were shown to possess flexible genome evolution to integrate genes from other individuals (Zhang et al., 2016), which may explain why such similar genotypes were found at a small scale.

The HTS-based approach used in this study demonstrated a high diversity of *Microcystis* genotypes coexisting in two water areas in Tianjin. Compared with traditional library cloning method, HTS provides more information for the comprehensive detection of environmental samples. With the development of HTS, additional advantages will be demonstrated. Future studies could monitor different proportions of each genotype during a bloom. The convenient and advanced method will provide the possibility to study diversity in *Microcystis* in larger scales.

## Conclusion

Using the newly generated primer for detecting *Microcystis* ITS region by HTS, we examined *Microcystis* intra-species diversity along a Yuqiao-Haihe water system in Tianjin, China. The results demonstrated an extremely higher diversity of *Microcystis* in studied waters compared with that in previous studies using the traditional cloning sequencing. The ITS-based genetic diversity of *Microcystis* populations was negatively related to eutrophication levels, and the dominant genotype in two water areas was mostly affected by TN and TP. Finally, phylogenetic analyses revealed a possible flexible genotype evolution at a small scale at Yuqiao-Haihe water system.

## Author Contributions

ZQ and RL designed the study. DH, YC, TZ, XL, and XZ performed the sampling and data analysis. DH wrote the manuscript. GY revised the manuscript and performed the primer checking experiment.

## Conflict of Interest Statement

The authors declare that the research was conducted in the absence of any commercial or financial relationships that could be construed as a potential conflict of interest.

## Supplementary Material

FIGURE S1 Length distribution of reads.

TABLE S1 Strains used for examination of *Microcysits*-specific ITS primers.

TABLE S2 Online BLAST based primer checking results (OTUs with abundance>10 is involved).

TABLE S3 Correlations between OTU abundance and environmental factors.
