# Supplementary figures and images for: Characterization of Microcystis (Cyanobacteria) Genotypes Based on the Internal Transcribed Spacer Region of rRNA by Next-Generation Sequencing
Source: Front Microbiol. 2018 May 15;9:971. doi: 10.3389/fmicb.2018.00971 (PMC5962762; doi:10.3389/fmicb.2018.00971)

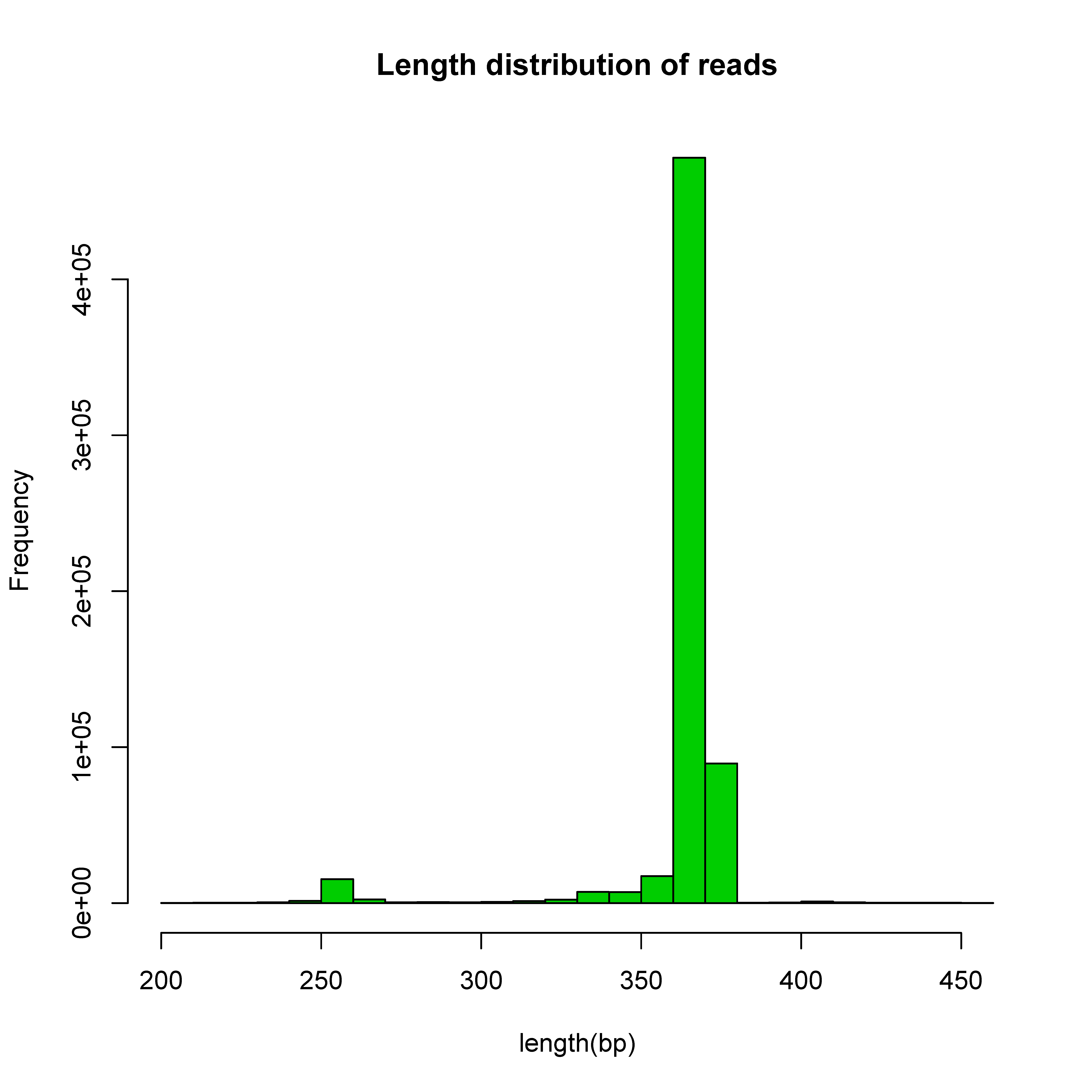

Supplement: FIGURE S1 — Length distribution of reads. [file Image_1.TIFF]
