# Supplementary material for: Characterization of Microcystis (Cyanobacteria) Genotypes Based on the Internal Transcribed Spacer Region of rRNA by Next-Generation Sequencing
Source: Front Microbiol. 2018 May 15;9:971. doi: 10.3389/fmicb.2018.00971 (PMC5962762; doi:10.3389/fmicb.2018.00971)
Supplement: TABLE S1 — Strains used for examination of Microcysits-specific ITS primers. [file Table_1.DOCX]

Supplementary S1 Strains used for examination of *Microcysits*-specific ITS primers

| Strain Name | Strains name | Results |
| --- | --- | --- |
| CHAB5926  NIES81  CHAB1039 | *Aphanizomenon* sp.  *Aphanizomenon flos-aquae*  *Aphanizomenon gracile* | -  -  - |
| CHAB2417 | *Cuspidothrix* sp. | - |
| CHAB*7031* | *Planktothrix* sp. | - |
| CHAB7029 | *Planktothricoides* sp. | - |
| CHAB7033  CHAB1629  CHAB3438  CHAB3409  CHAB3422 | *Dolichospermum* sp.  *Dolichospermum* sp.  *Cylindrospermopsis raciborskii*  *Cylindrospermopsis raciborskii*  *Cylindrospermopsis raciborskii* | -  -  -  -  - |
| CHAB2801  CHAB5733  CHAB5712  CHAB5766  CHAB1147  CHAB796  CHAB7039  CHAB1290  CHAB1291  CHAB7000  CHAB1289  CHAB1283 | *Nostoc sp.*  *Nostoc sp.*  *Nostoc sp.*  *Nostoc sp.*  *Pseudanabaena mucicola*  *Pseudanabaena limnetica*  *Limnothrix* sp.  Lyngbya sp.  Lyngbya sp.  *Oscillatoria* sp.  *Synechococcus*  *Synechococcus* | -  -  -  -  -  -  -  -  -  -  -  - |
| CHAB7021 | *Merismopedia* | - |
| PCC7806 | *Microcysits* sp. | + |
| CHAB7032 | *Microcysits* sp. | + |
| NIES-843  CHAB6305  CHAB6307  CHAB6309  CHAB6317  CHAB6318  CHAB6366  CHAB6369  CHAB6372  CHAB6383  CHAB6387  CHAB6388  CHAB6328 CHAB6330  CHAB6331  CHAB6334  CHAB6338  CHAB6343  CHAB6348  CHAB6353  CHAB6357  CHAB6370  CHAB6374  CHAB6378  CHAB6380  CHAB329  CHAB331  CHAB3381  CHAB3384  CHAB3388 | *Microcysits* sp.  *Microcysits wesenbergii*  *Microcysits wesenbergii*  *Microcysits wesenbergii*  *Microcysits wesenbergii*  *Microcysits wesenbergii*  *Microcysits viridis*  *Microcysits viridis*  *Microcysits viridis*  *Microcysits viridis*  *Microcysits viridis*  *Microcysits viridis*  *Microcysits aeruginosa*  *Microcysits aeruginosa*  *Microcysits aeruginosa*  *Microcysits aeruginosa*  *Microcysits aeruginosa*  *Microcysits aeruginosa*  *Microcysits aeruginosa*  *Microcysits aeruginosa*  *Microcysits aeruginosa*  *Microcysits aeruginosa*  *Microcysits aeruginosa*  *Microcysits aeruginosa*  *Microcysits aeruginosa*  *Microcysits botrys*  *Microcysits botrys*  *Microcysits botrys*  *Microcysits botrys*  *Microcysits botrys* | +  +  +  +  +  +  +  +  +  +  +  +  +  +  +  +  +  +  +  +  +  +  +  +  +  +  +  +  +  + |
| Negative Control | *-* | - |
