# Supplementary material for: Characterization of Microcystis (Cyanobacteria) Genotypes Based on the Internal Transcribed Spacer Region of rRNA by Next-Generation Sequencing
Source: Front Microbiol. 2018 May 15;9:971. doi: 10.3389/fmicb.2018.00971 (PMC5962762; doi:10.3389/fmicb.2018.00971)
Supplement: TABLE S2 — Online BLAST based primer checking results (OTUs with abundance>10 is involved). [file Table_2.DOCX]

Online BLAST based primer checking results (OTUs with abundance>10 is involved)

| Blast-hit results | Abundance | Percentage |
| --- | --- | --- |
| Microcystis | 517339 | 99.29% |
| Unclassified | 3174 | 0.61% |
| No-hit | 359 | 0.07% |
| Limnothrix | 51 | 0.01% |
| Limnohabitans | 32 | 0.01% |
| Phormidium | 26 | 0.00% |
| Cylindrospermopsis | 11 | 0.00% |
| Shigella | 11 | 0.00% |
| Aeromonas | 10 | 0.00% |
| Pseudomonas | 10 | 0.00% |
