# Supplementary material for: Characterization of Microcystis (Cyanobacteria) Genotypes Based on the Internal Transcribed Spacer Region of rRNA by Next-Generation Sequencing
Source: Front Microbiol. 2018 May 15;9:971. doi: 10.3389/fmicb.2018.00971 (PMC5962762; doi:10.3389/fmicb.2018.00971)
Supplement: TABLE S3 — Correlations between OTU abundance and environmental factors. [file Table_3.DOCX]

|  | TP_VALUE  cor | TP_VALUE  P_value | NH4  cor | NH4  P_value | NO2  cor | NO2  P_value | NO3  cor | NO3  P_value | COD  cor | COD  P_value | TN  cor | TN  P_value | chl  cor | chl  P_value |
| --- | --- | --- | --- | --- | --- | --- | --- | --- | --- | --- | --- | --- | --- | --- |
| Otu0 | 0.57 | 0.03 | 0.02 | 0.95 | 0.43 | 0.11 | -0.36 | 0.19 | 0.19 | 0.50 | 0.59 | 0.02 | 0.43 | 0.11 |
| Otu4 | 0.02 | 0.94 | -0.43 | 0.11 | -0.44 | 0.10 | -0.57 | 0.03 | 0.62 | 0.01 | 0.15 | 0.59 | 0.29 | 0.29 |
| Otu2 | -0.40 | 0.14 | 0.23 | 0.40 | -0.08 | 0.79 | 0.52 | 0.05 | -0.59 | 0.02 | -0.40 | 0.14 | -0.48 | 0.07 |
| Otu8 | -0.11 | 0.69 | -0.46 | 0.09 | -0.13 | 0.63 | -0.18 | 0.51 | 0.28 | 0.31 | -0.11 | 0.70 | -0.02 | 0.93 |
| Otu1 | -0.08 | 0.77 | -0.30 | 0.27 | -0.32 | 0.25 | -0.26 | 0.36 | 0.40 | 0.14 | -0.07 | 0.81 | 0.28 | 0.31 |
| Otu5 | 0.06 | 0.84 | -0.24 | 0.39 | -0.34 | 0.21 | -0.32 | 0.24 | 0.35 | 0.20 | 0.14 | 0.62 | 0.30 | 0.27 |
| Otu3 | -0.15 | 0.59 | 0.20 | 0.47 | 0.20 | 0.48 | 0.37 | 0.18 | -0.58 | 0.02 | -0.23 | 0.41 | -0.33 | 0.23 |
| Otu6 | 0.01 | 0.96 | -0.20 | 0.48 | -0.37 | 0.17 | -0.54 | 0.04 | 0.57 | 0.03 | 0.14 | 0.62 | 0.10 | 0.71 |
| Otu14 | 0.18 | 0.52 | 0.72 | 0.00 | 0.70 | 0.00 | 0.53 | 0.04 | -0.56 | 0.03 | 0.07 | 0.82 | -0.14 | 0.62 |
| Otu15 | -0.20 | 0.47 | 0.15 | 0.61 | 0.24 | 0.38 | 0.75 | 0.00 | -0.63 | 0.01 | -0.36 | 0.19 | -0.45 | 0.09 |
| Otu12 | 0.50 | 0.06 | 0.48 | 0.07 | 0.48 | 0.07 | 0.10 | 0.72 | -0.29 | 0.29 | 0.40 | 0.14 | 0.35 | 0.20 |
| Otu7 | -0.08 | 0.77 | -0.59 | 0.02 | -0.16 | 0.57 | -0.43 | 0.11 | 0.29 | 0.29 | 0.21 | 0.46 | 0.02 | 0.93 |
| Otu10 | -0.36 | 0.18 | -0.04 | 0.89 | -0.14 | 0.63 | 0.42 | 0.12 | -0.63 | 0.01 | -0.36 | 0.18 | -0.47 | 0.08 |
| Otu293 | -0.39 | 0.15 | -0.12 | 0.68 | -0.26 | 0.35 | 0.57 | 0.03 | -0.52 | 0.04 | -0.41 | 0.13 | -0.38 | 0.17 |
| Otu16 | 0.17 | 0.53 | 0.53 | 0.04 | 0.43 | 0.11 | 0.55 | 0.03 | -0.64 | 0.01 | 0.02 | 0.94 | -0.09 | 0.75 |
| Otu279 | 0.05 | 0.86 | 0.19 | 0.49 | -0.16 | 0.58 | -0.27 | 0.33 | 0.26 | 0.35 | 0.15 | 0.59 | 0.02 | 0.93 |
| Otu9 | -0.21 | 0.45 | -0.17 | 0.55 | 0.01 | 0.97 | 0.58 | 0.02 | -0.56 | 0.03 | -0.38 | 0.16 | -0.38 | 0.16 |
| Otu11 | -0.28 | 0.31 | 0.23 | 0.41 | -0.09 | 0.74 | 0.36 | 0.19 | -0.55 | 0.03 | -0.26 | 0.36 | -0.38 | 0.16 |
| Otu280 | -0.34 | 0.22 | -0.13 | 0.65 | -0.29 | 0.29 | 0.51 | 0.05 | -0.41 | 0.13 | -0.35 | 0.20 | -0.30 | 0.28 |
| Otu13 | -0.27 | 0.32 | -0.54 | 0.04 | -0.14 | 0.61 | -0.08 | 0.77 | 0.05 | 0.85 | -0.23 | 0.40 | -0.23 | 0.40 |

|  | WT  cor | WT  P_value | DO  cor | DO  P_value | SAL  cor | SAL  P_value | P_valueH  cor | P_valueH  P_value |
| --- | --- | --- | --- | --- | --- | --- | --- | --- |
| Otu0 | -0.24 | 0.39 | 0.13 | 0.66 | 0.37 | 0.17 | 0.38 | 0.16 |
| Otu4 | 0.31 | 0.27 | -0.77 | 0.00 | -0.59 | 0.02 | -0.41 | 0.13 |
| Otu2 | -0.02 | 0.95 | 0.64 | 0.01 | 0.07 | 0.81 | -0.01 | 0.97 |
| Otu8 | -0.40 | 0.14 | -0.31 | 0.26 | -0.39 | 0.15 | -0.23 | 0.40 |
| Otu1 | -0.23 | 0.42 | -0.38 | 0.16 | -0.27 | 0.33 | -0.22 | 0.42 |
| Otu5 | 0.33 | 0.23 | -0.47 | 0.08 | -0.38 | 0.16 | -0.35 | 0.20 |
| Otu3 | -0.01 | 0.98 | 0.38 | 0.16 | 0.26 | 0.35 | 0.16 | 0.57 |
| Otu6 | 0.42 | 0.12 | -0.68 | 0.01 | -0.56 | 0.03 | -0.29 | 0.30 |
| Otu14 | -0.15 | 0.59 | 0.72 | 0.00 | 0.76 | 0.00 | 0.49 | 0.06 |
| Otu15 | -0.21 | 0.46 | 0.56 | 0.03 | 0.32 | 0.25 | 0.30 | 0.27 |
| Otu12 | -0.17 | 0.54 | 0.78 | 0.00 | 0.84 | 0.00 | 0.68 | 0.01 |
| Otu7 | -0.01 | 0.97 | -0.45 | 0.09 | -0.37 | 0.17 | -0.29 | 0.29 |
| Otu10 | -0.06 | 0.84 | 0.50 | 0.06 | -0.09 | 0.74 | -0.05 | 0.86 |
| Otu293 | -0.01 | 0.98 | 0.36 | 0.18 | 0.21 | 0.46 | 0.14 | 0.61 |
| Otu16 | -0.12 | 0.66 | 0.92 | 0.00 | 0.70 | 0.00 | 0.60 | 0.02 |
| Otu279 | 0.57 | 0.03 | -0.28 | 0.30 | -0.19 | 0.50 | 0.10 | 0.72 |
| Otu9 | 0.03 | 0.92 | 0.34 | 0.21 | 0.07 | 0.81 | 0.17 | 0.54 |
| Otu11 | 0.01 | 0.98 | 0.50 | 0.06 | -0.09 | 0.76 | -0.04 | 0.87 |
| Otu280 | -0.03 | 0.92 | 0.35 | 0.20 | 0.19 | 0.50 | 0.13 | 0.64 |
| Otu13 | -0.41 | 0.13 | -0.11 | 0.69 | -0.25 | 0.36 | -0.33 | 0.23 |
